# Supplementary material for: Novel Perspectives on the Design and Development of a Long-Acting Subcutaneous Raltegravir Injection for Treatment of HIV—In Vitro and In Vivo Evaluation
Source: Pharmaceutics. 2023 May 18;15(5):1530. doi: 10.3390/pharmaceutics15051530 (PMC10224444; doi:10.3390/pharmaceutics15051530)
Supplement: Supplementary file 1 [file pharmaceutics-15-01530-s001.zip › pharmaceutics-2366990-supplementary.pdf]

Novel Perspectives on the Design and Development of a Long-Acting Subcutaneous Raltegravir Injection for Treatment of HIV—In Vitro and In Vivo Evaluation

Heba S. Abd-Ellah [1,2], Ramesh Mudududdla [1,*], Glen P. Carter [3] and Jonathan B. Baell [1,4,*]

[1] Medicinal Chemistry, Monash Institute of Pharmaceutical Sciences, Parkville, VIC 3052, Australia; hebasaleh.chem@gmail.com
[2] Medicinal Chemistry Department, Faculty of Pharmacy, Minia University, Minia 61519, Egypt
[3] Microbiology and Immunology Department, Peter Doherty Institute for Infection and Immunity, The University of Melbourne, Parkville, VIC 3001, Australia; glen.carter@unimelb.edu.au
[4] School of Pharmaceutical Sciences, Nanjing Tech University, No. 30 South Puzhu Road, Nanjing 211816, China
* Correspondence: ramesh.mudududdla@monash.edu (R.M.); jonathan.baell@njtech.edu.cn or jbaell29@gmail.com (J.B.B.); Tel.: +61470330093 (R.M.); +61402194571 (J.B.B.)

Table of content

		Figures	Pages
NMR spectra of key intermediates **9a-b**, **10a-b**, **11a**, **13a-b, 19a-b,** and final probes **14-15, 20-21**		S1-S30	1-16
NMR spectra of intermediates **23**, **24**, side products **22**, **26** and prodrug **25**		S31-44	17-23

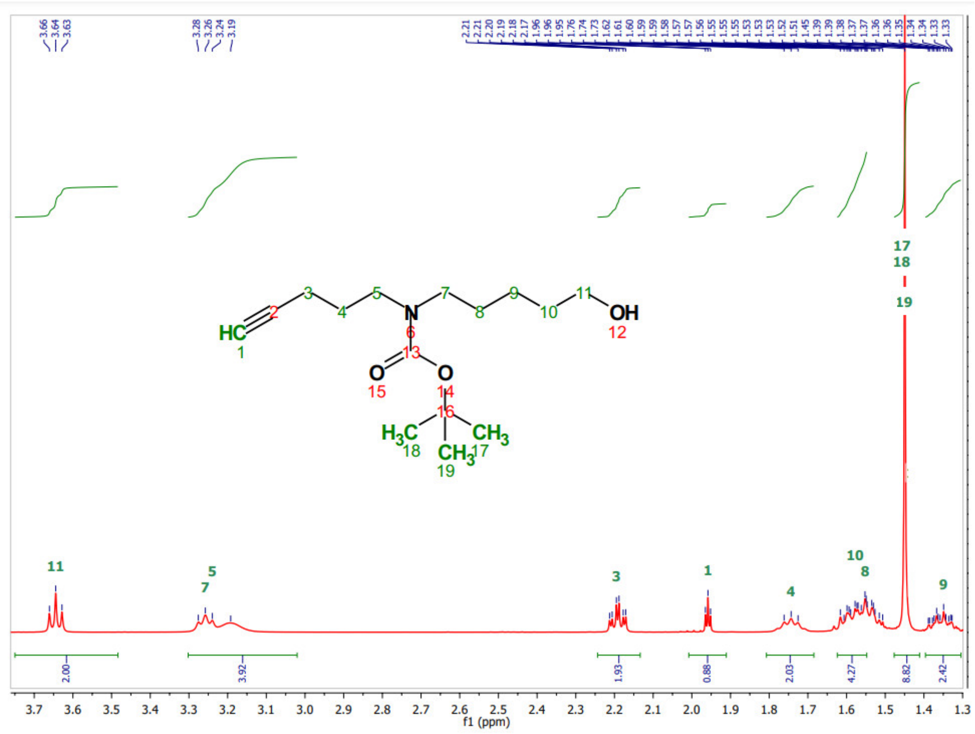

Figure S1. ^{1}H NMR of **9a** in $CDCl_3$.

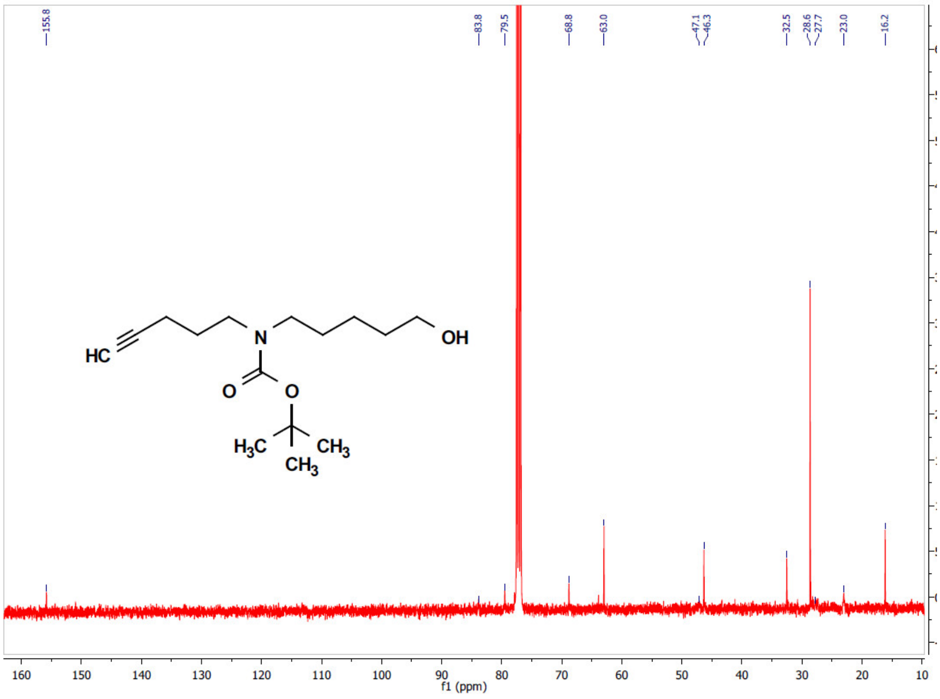

Figure S2. ^{13}C NMR of **9a** in $CDCl_3$.

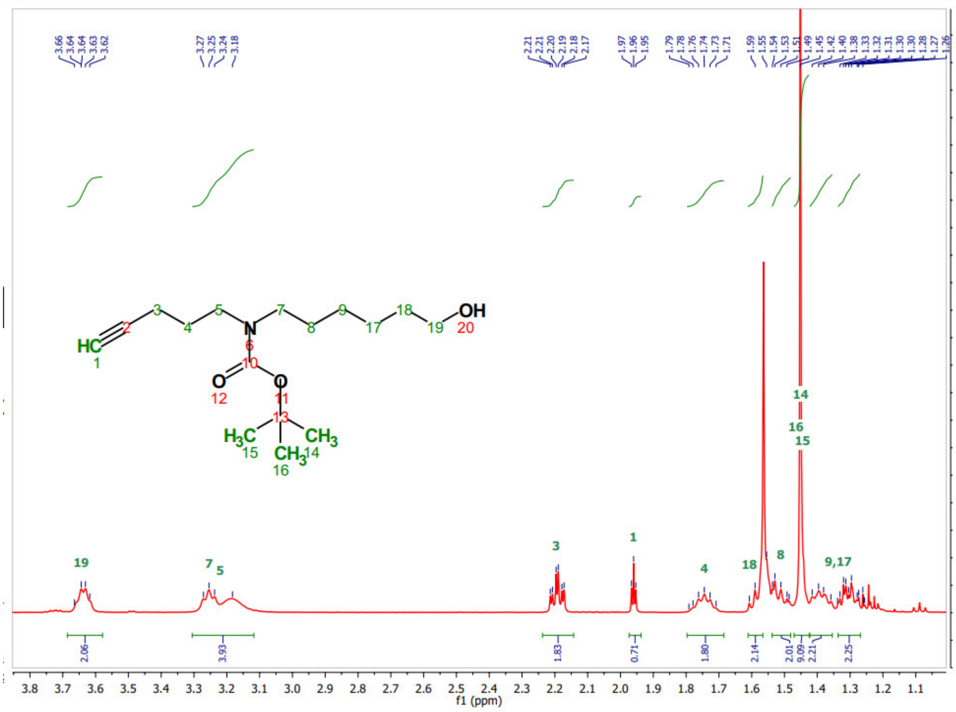

Figure S3. ^{1}H NMR of **9b** in $CDCl_3$.

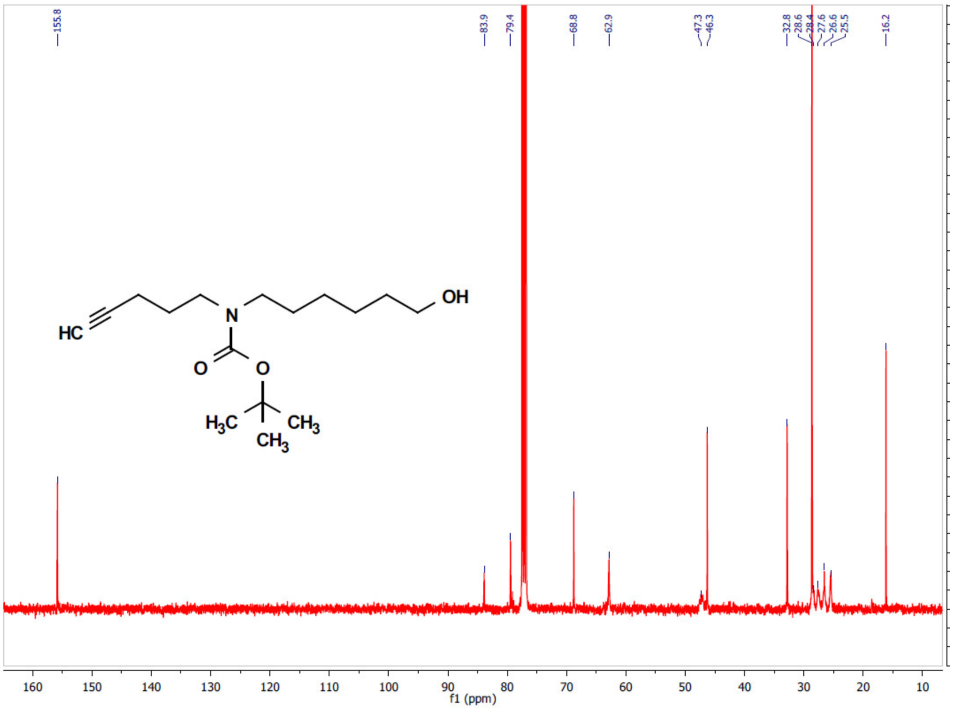

Figure S4. ^{13}C NMR of **9b** in $CDCl_3$.

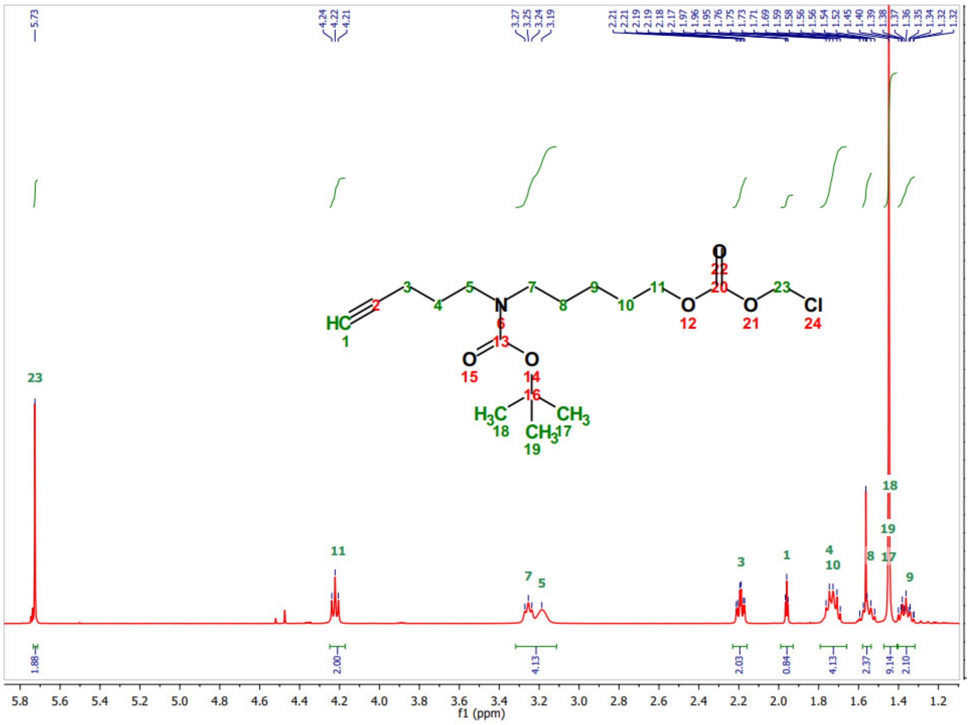

Figure S5. ^{1}H NMR of **10a** in $CDCl_3$.

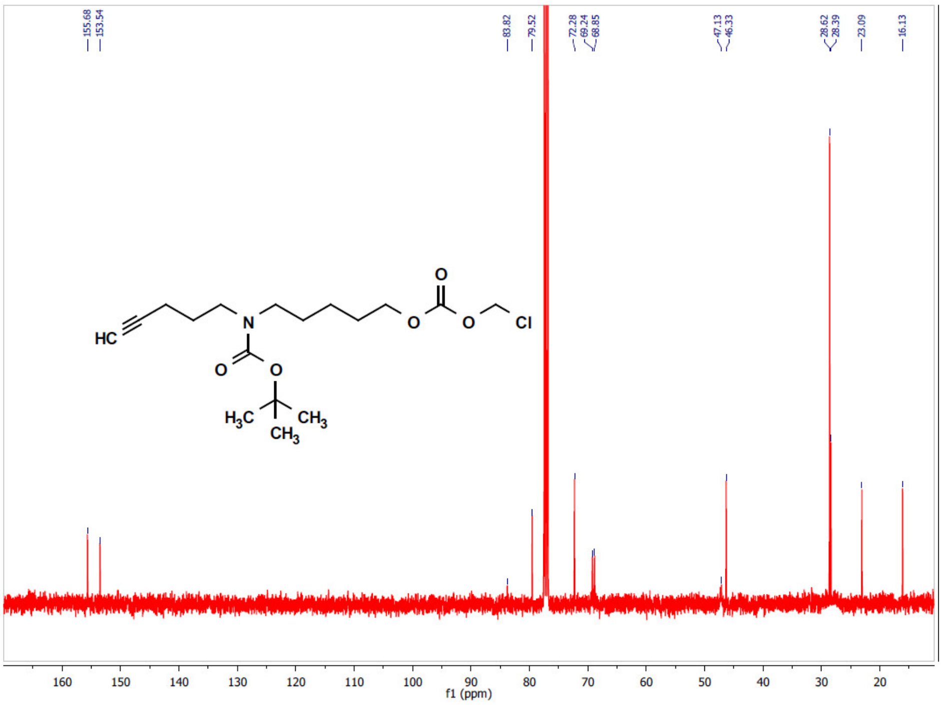

Figure S6. ^{13}C NMR of **10a** in $CDCl_3$.

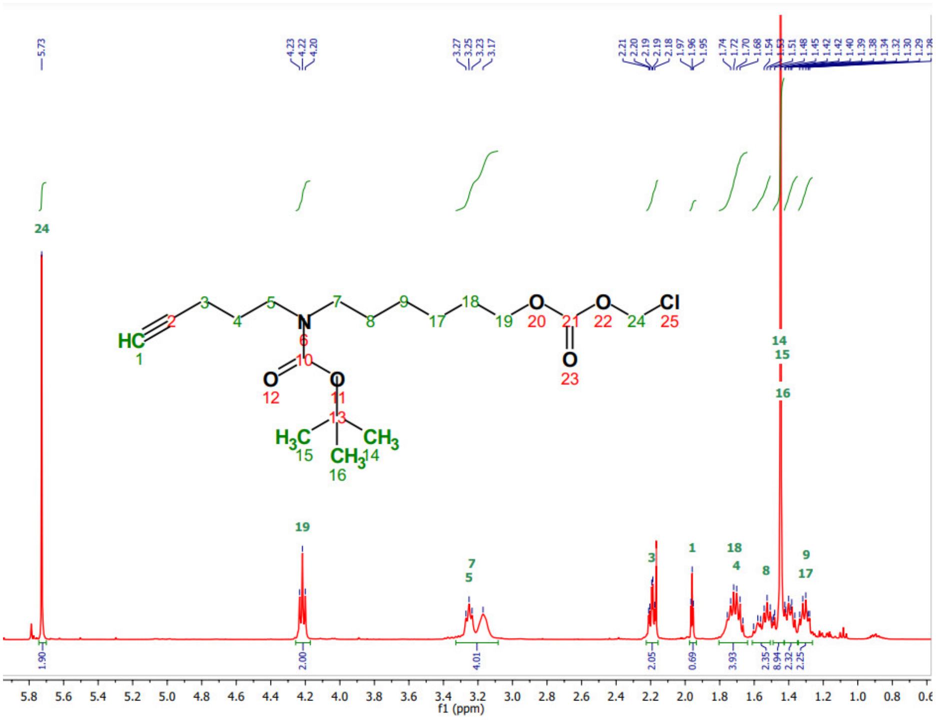

Figure S7. ^{1}H NMR of **10b** in $CDCl_3$.

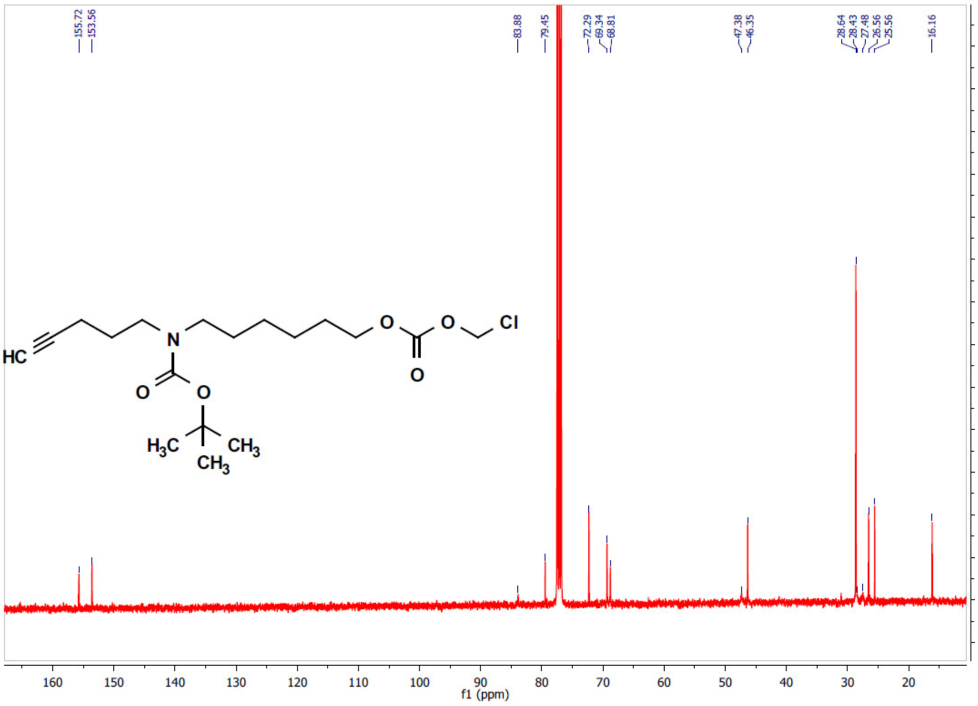

Figure S8. ^{13}C NMR of **10b** in $CDCl_3$.

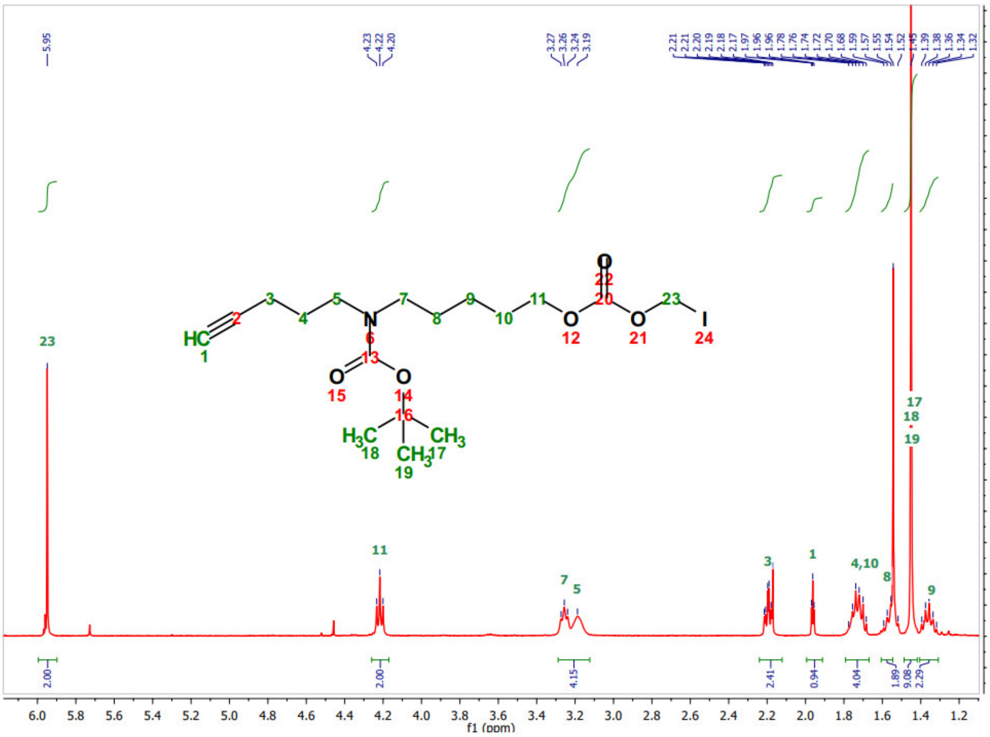

Figure S9. ^{1}H NMR of **11a** in $CDCl_3$.

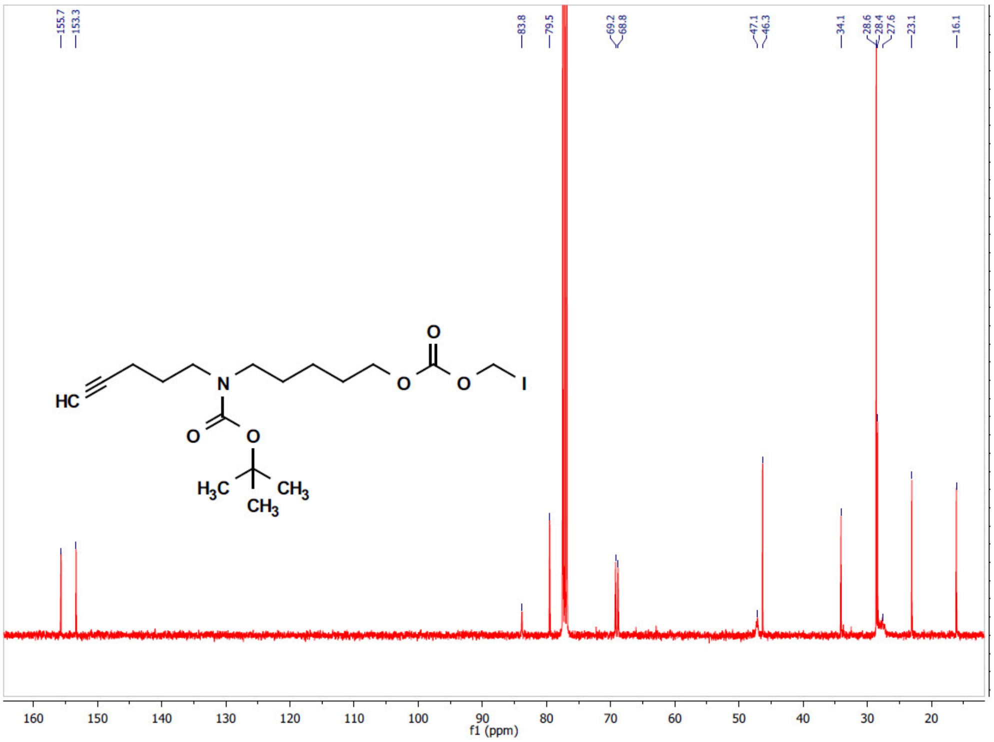

Figure S10. ^{13}C NMR of **11a** in $CDCl_3$.

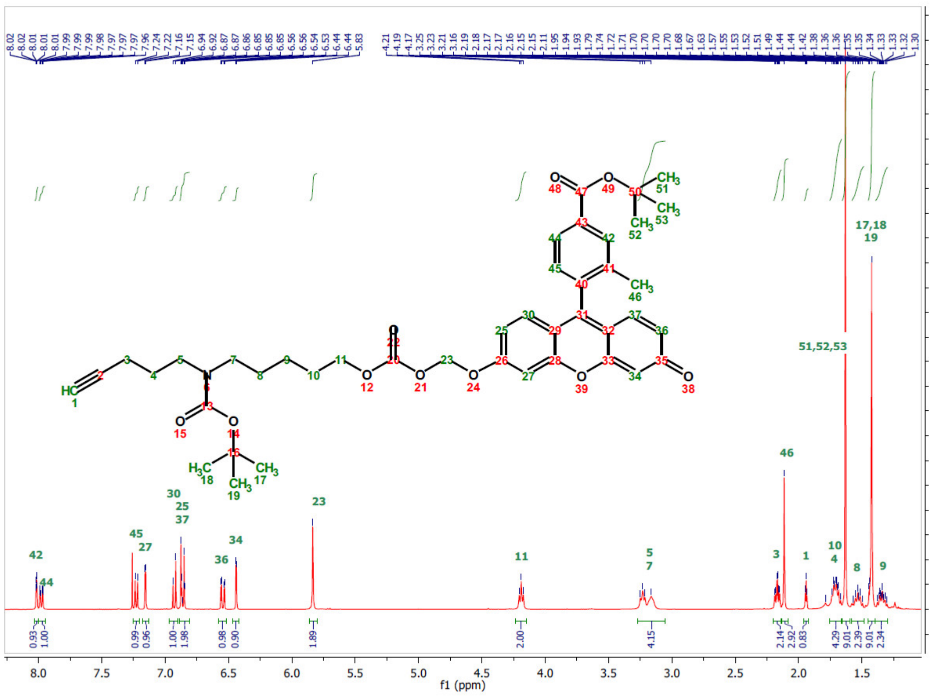

Figure S11. ^{1}H NMR of **13a** in $CDCl_3$.

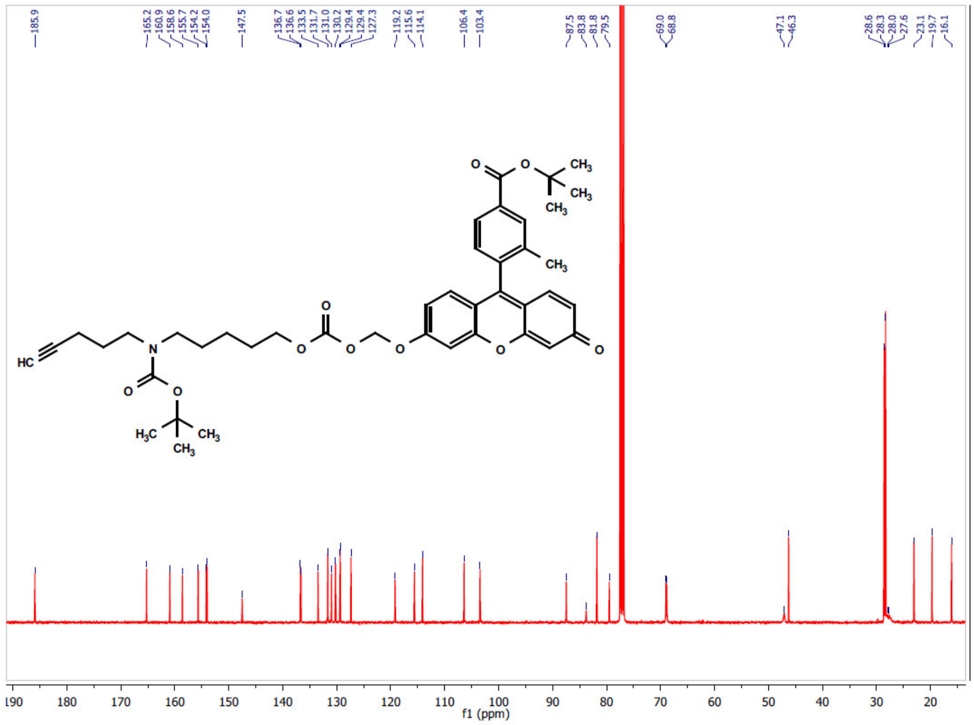

Figure S12. ^{13}C NMR of **13a** in $CDCl_3$.

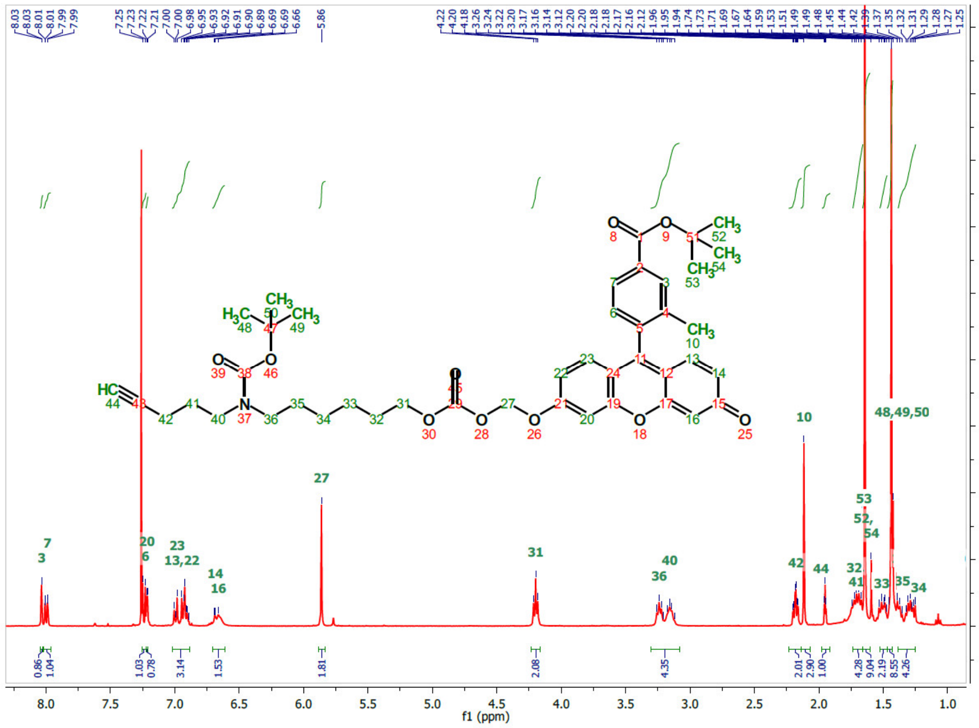

Figure S13. ^{1}H NMR of **13b** in $CDCl_3$.

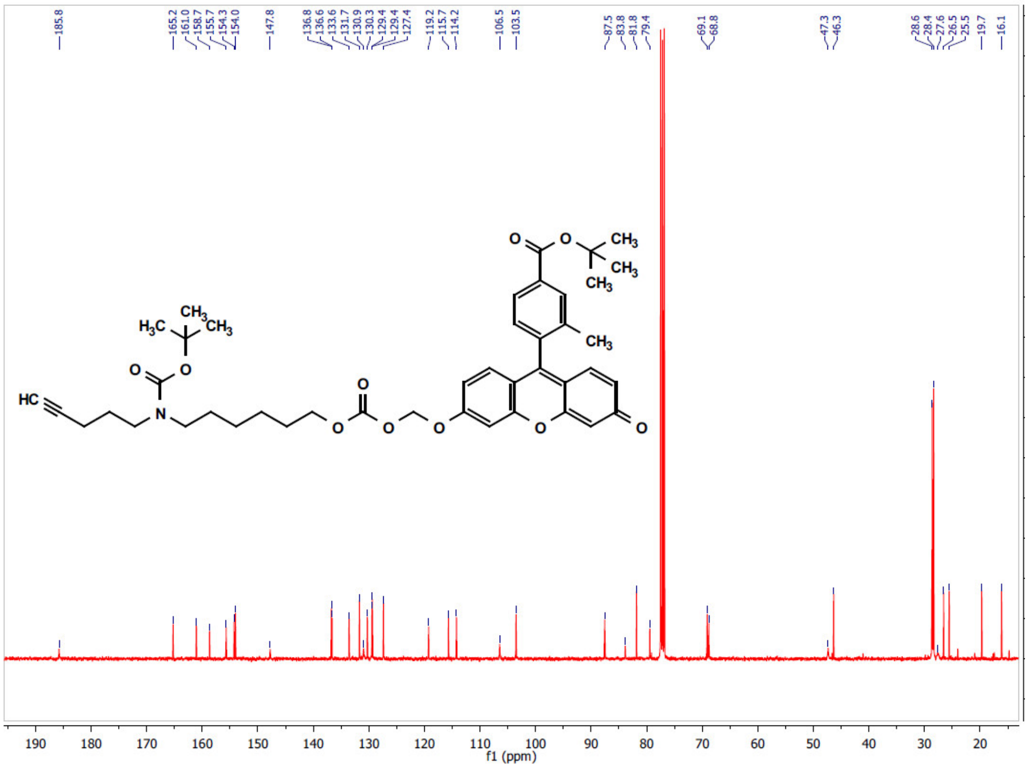

Figure S14. ^{13}C NMR of **13b** in $CDCl_3$.

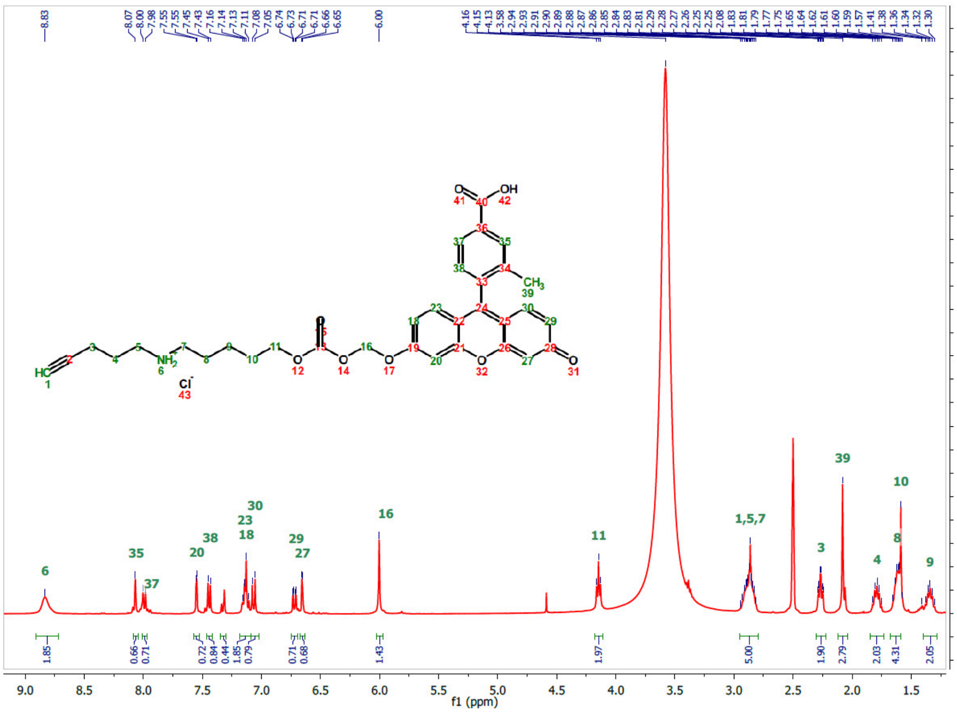

Figure S15. ^{1}H NMR of probe **14** in DMSO-d_6.

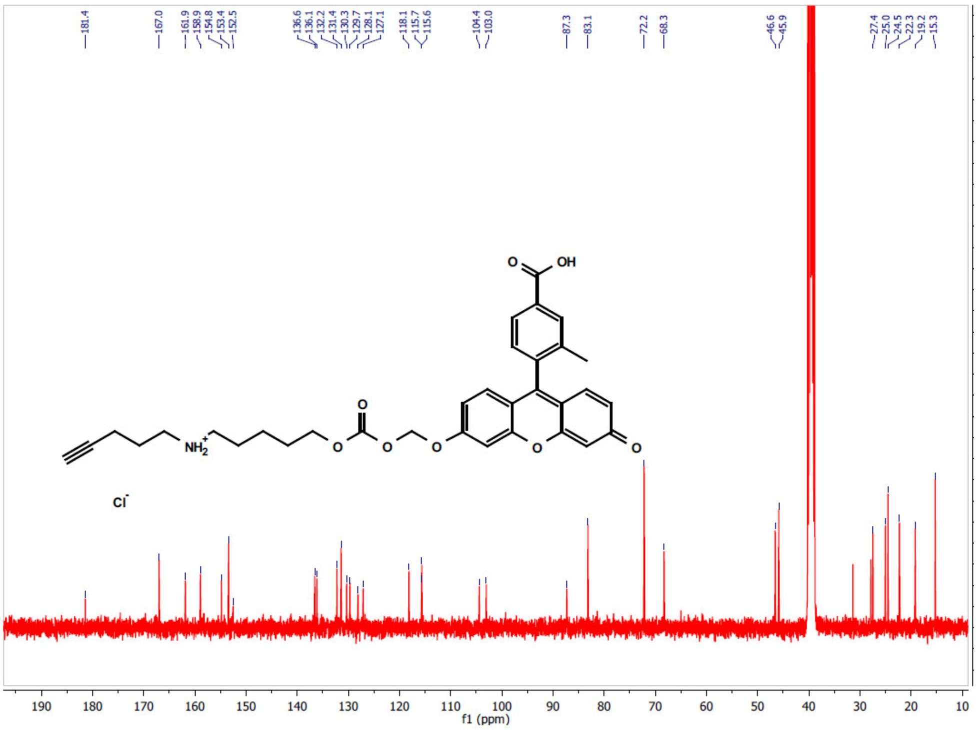

Figure S16. ^{13}C NMR of probe **14** in $CDCl_3$.

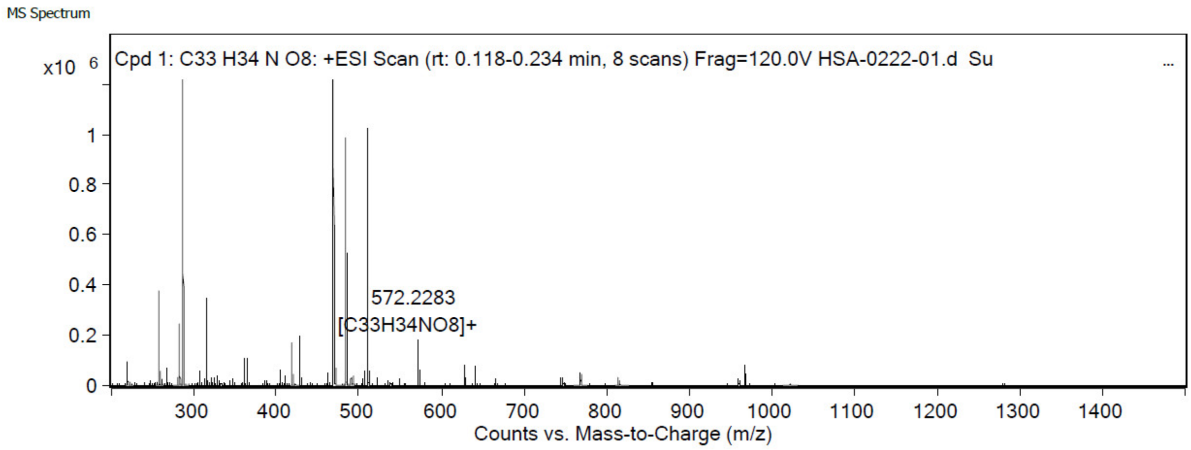

Figure S17. HRMS of probe **14**.

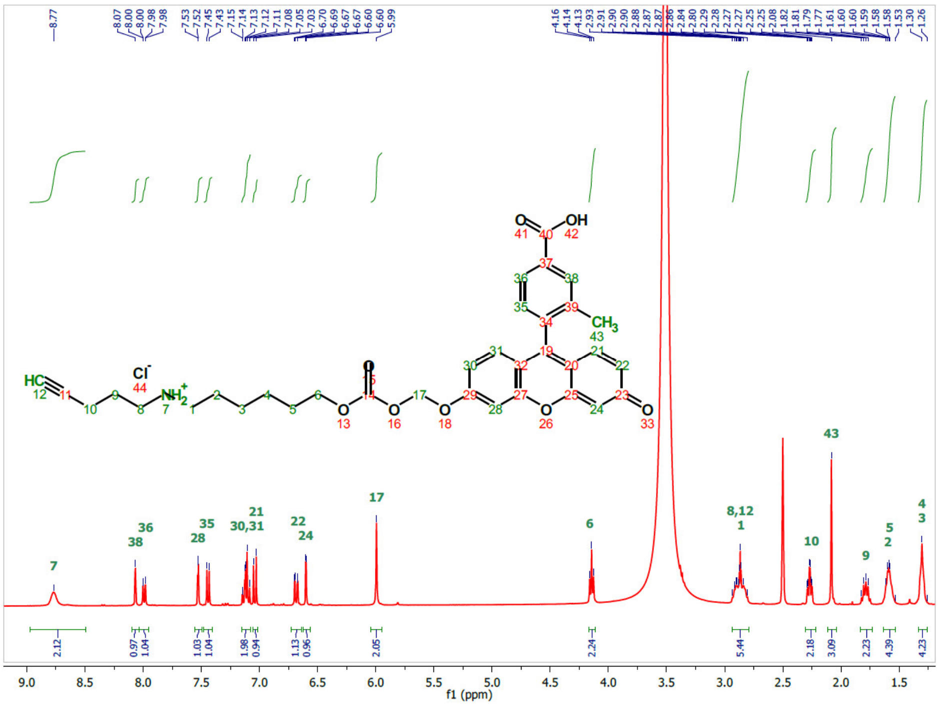

Figure S18. ^{1}H NMR of probe **15** in DMSO-d_6.

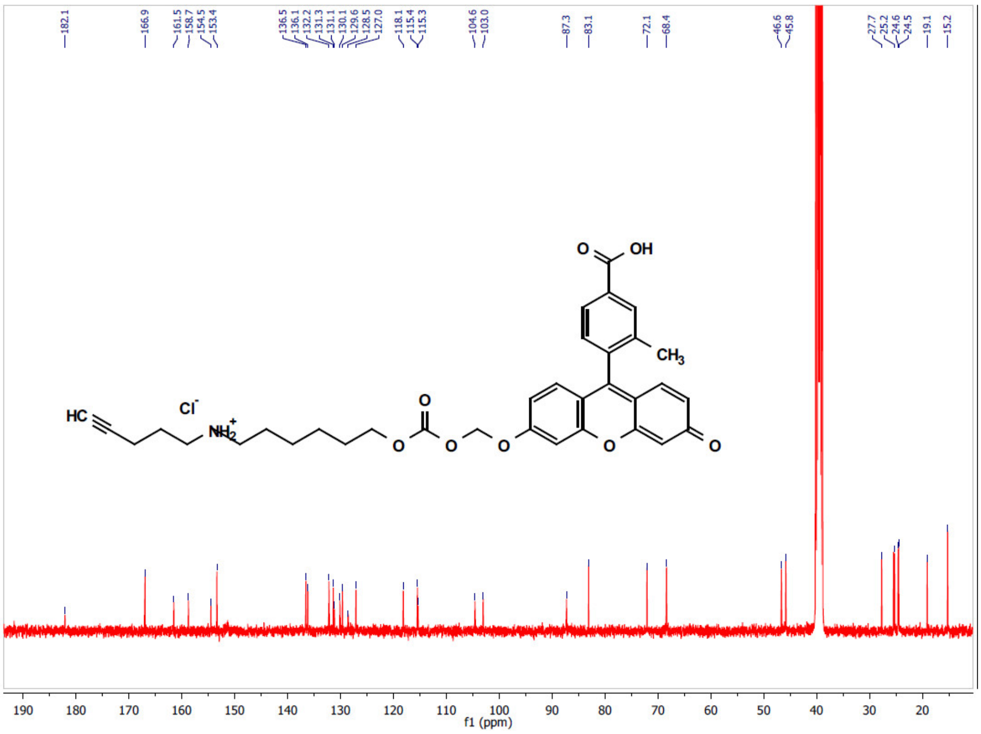

Figure S19. ^{13}C NMR of probe **15** in $CDCl_3$.

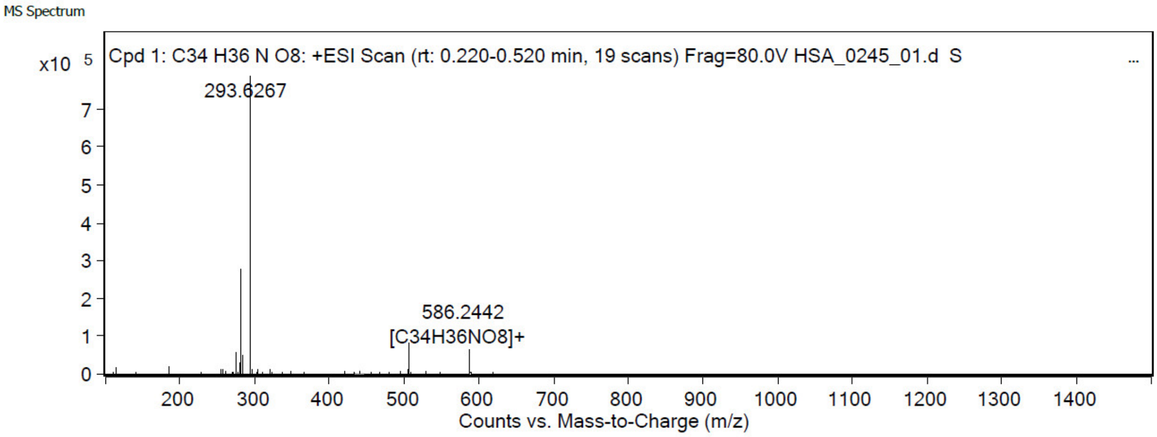

Figure S20. HRMS of probe **15**.

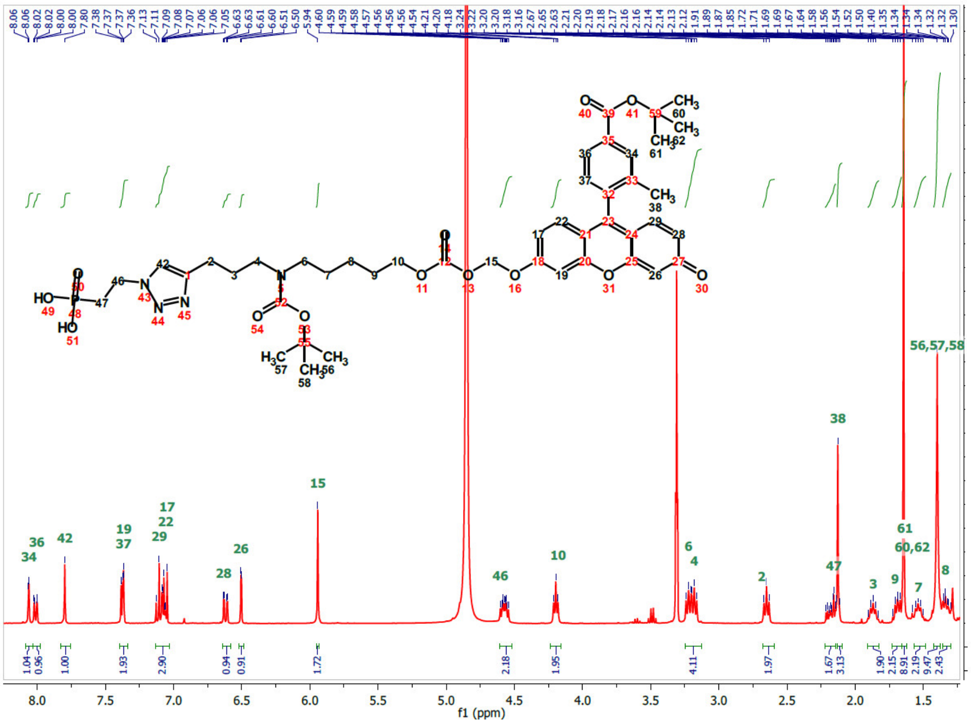

Figure S21. ^{1}H NMR of **19a** in MeOD.

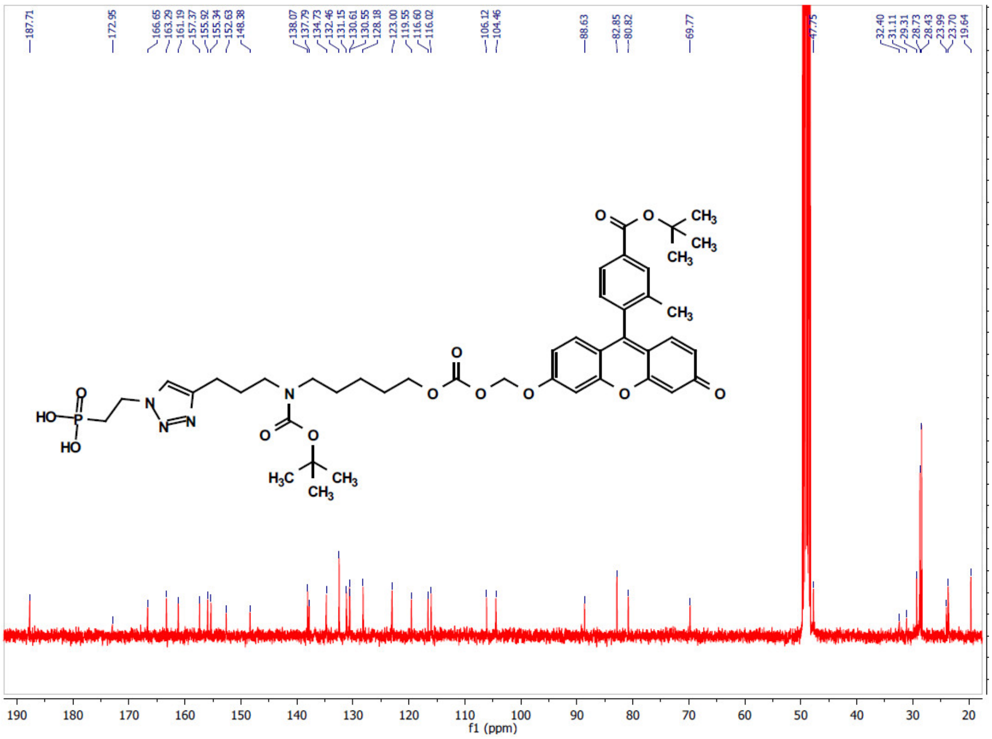

Figure S22. ^{13}C NMR of **19a** in MeOD.

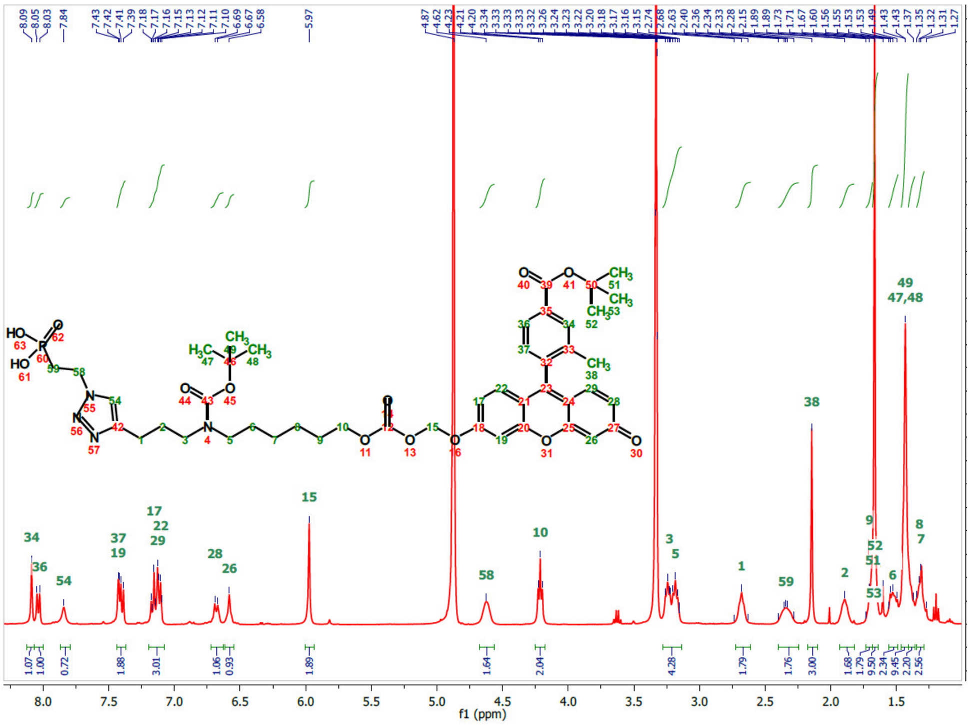

Figure S23. ^{1}H NMR of **19b** in MeOD.

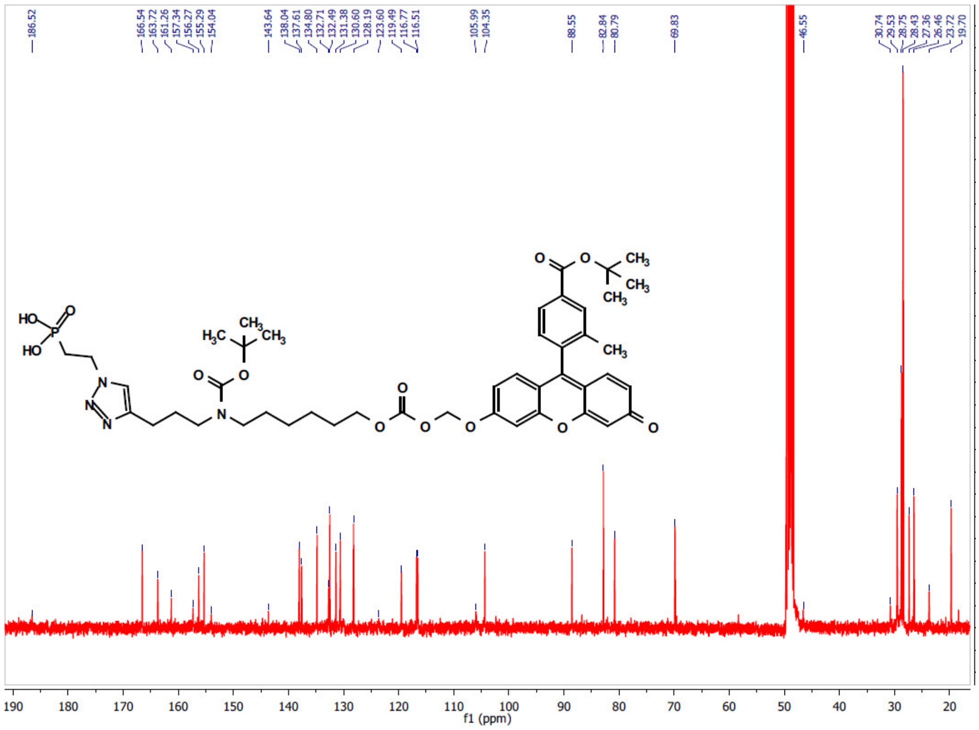

Figure S24. ^{13}C NMR of **19b** in MeOD.

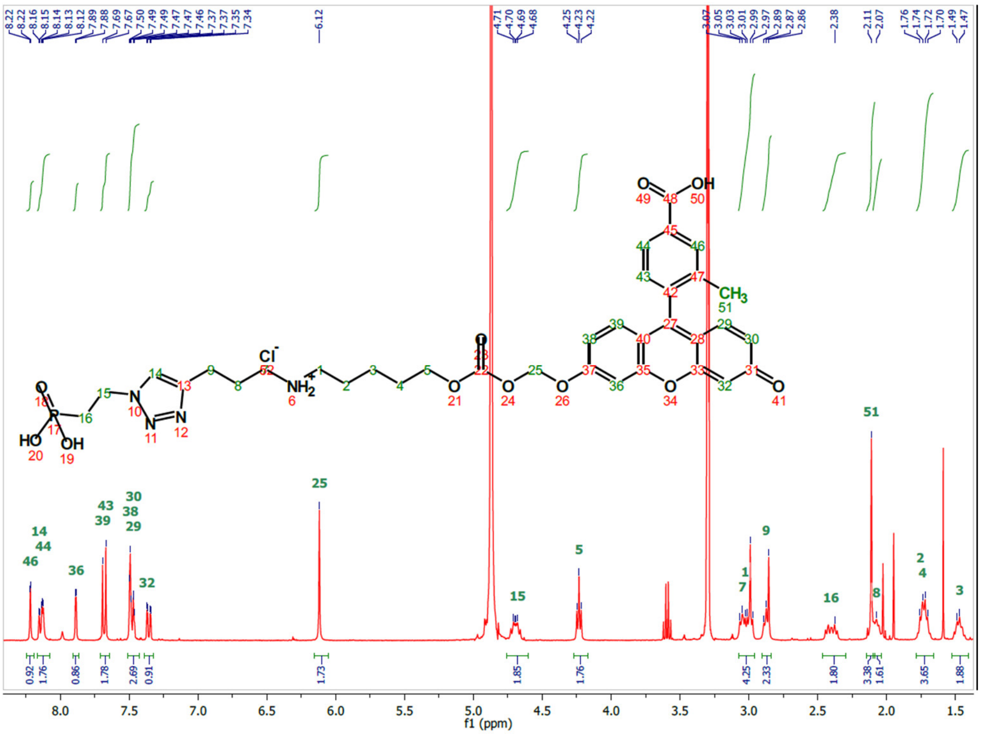

Figure S25. ^{1}H NMR of probe **20** in MeOD.

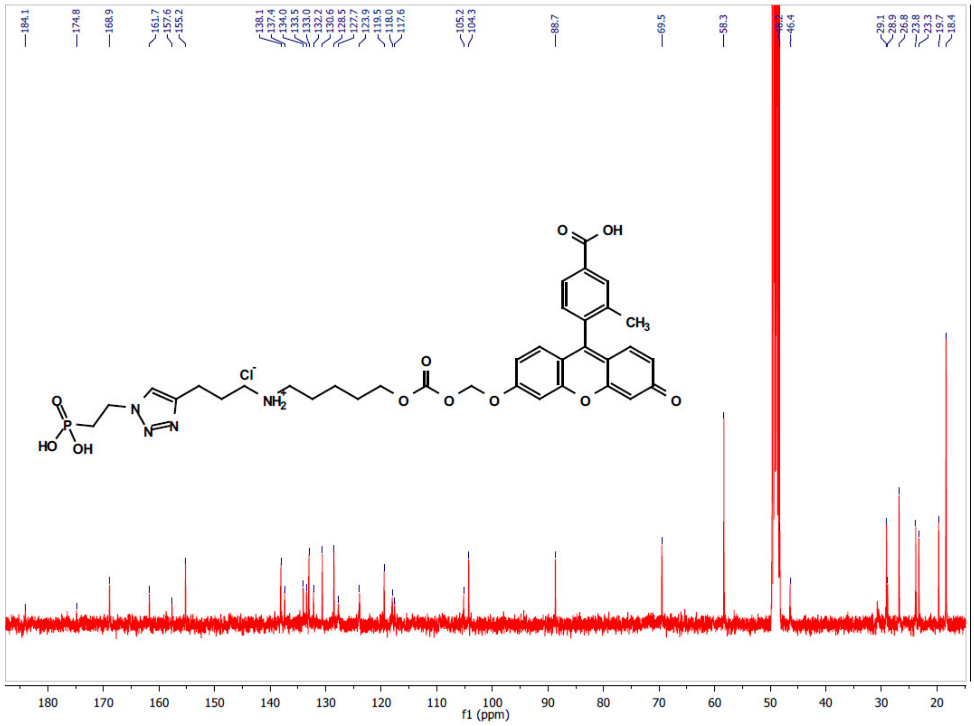

Figure S26. ^{13}C NMR of probe **20** in MeOD.

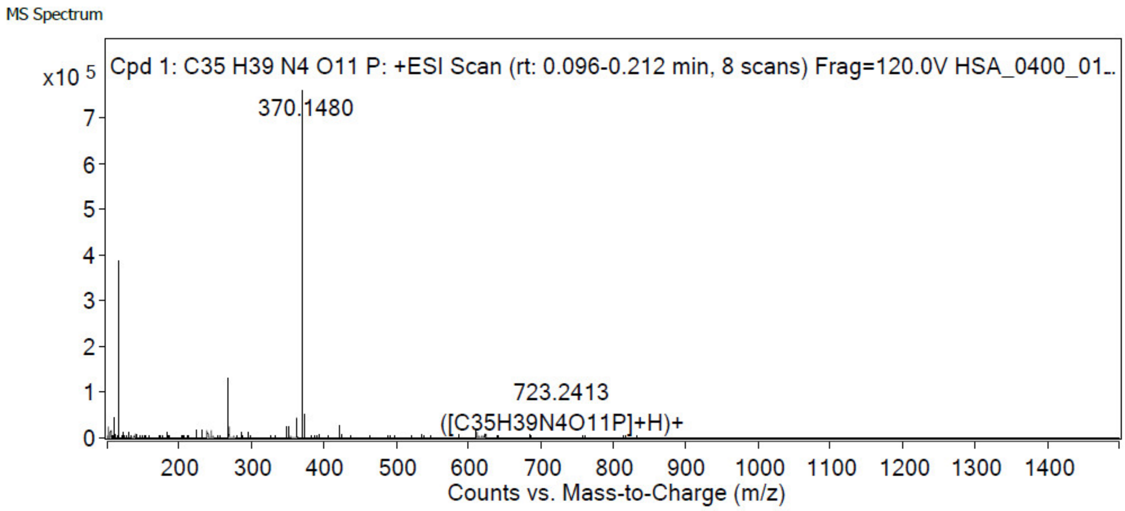

Figure S27. HRMS of probe **20**.

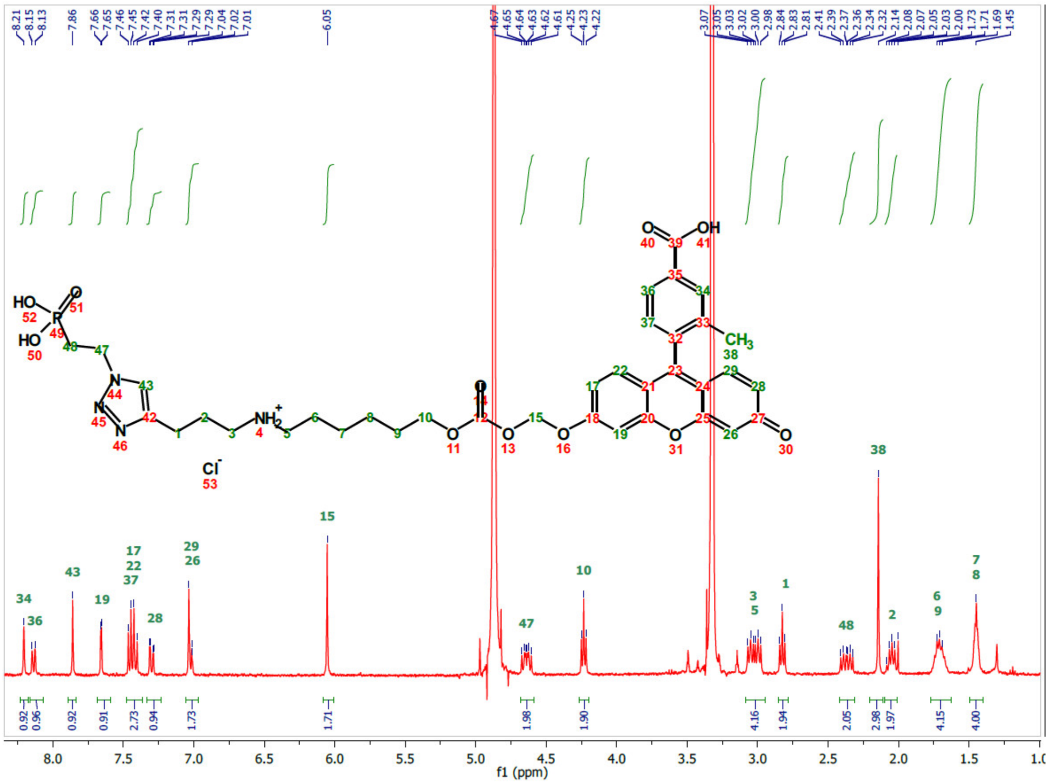

Figure S28. ^{1}H NMR of probe **21** in MeOD.

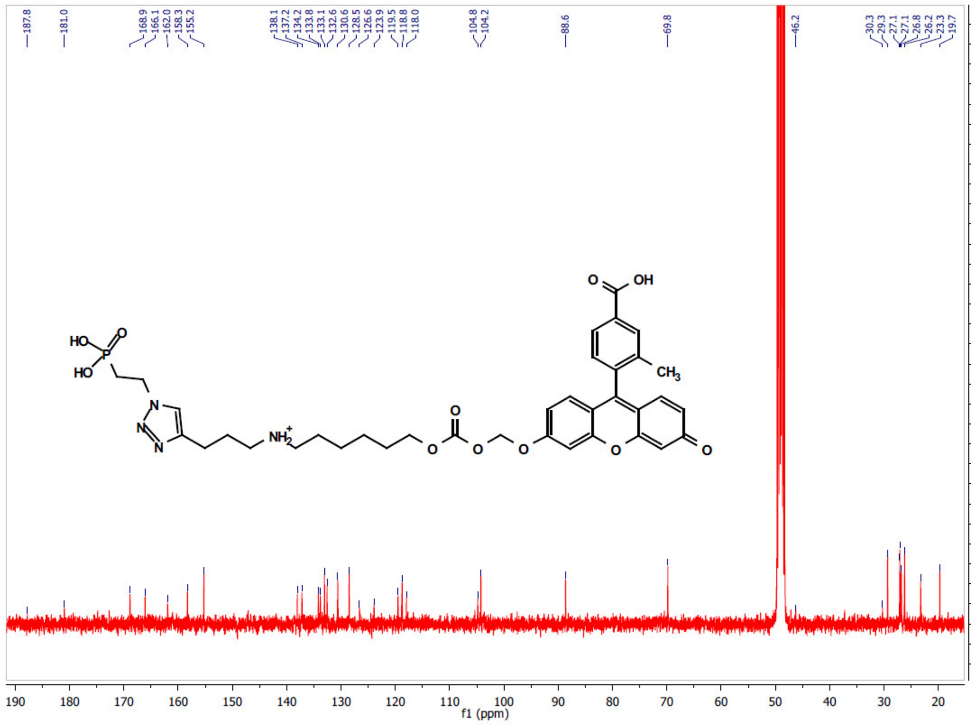

Figure S29. ^{13}C NMR of probe **21** in MeOD.

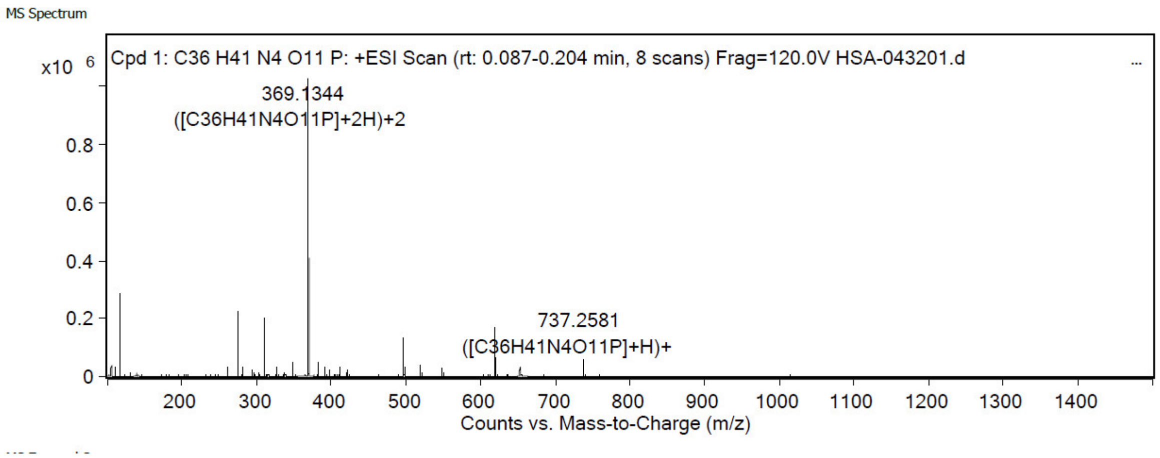

Figure S30. HRMS of probe **21**.

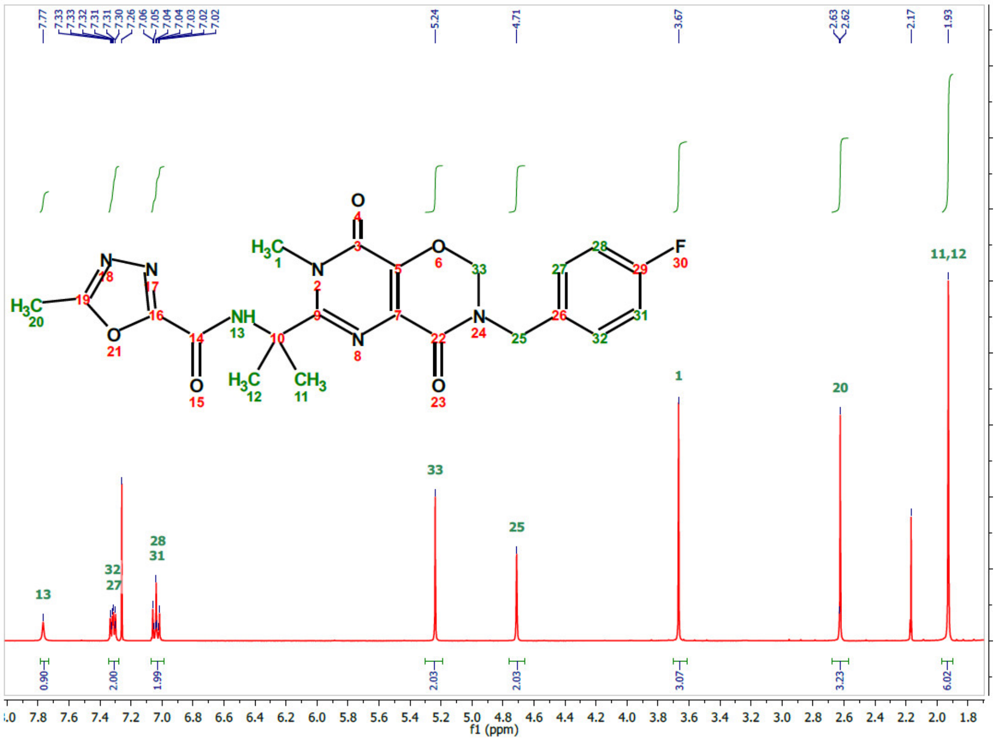

Figure S31. ^{1}H NMR of **22** in $CDCl_3$.

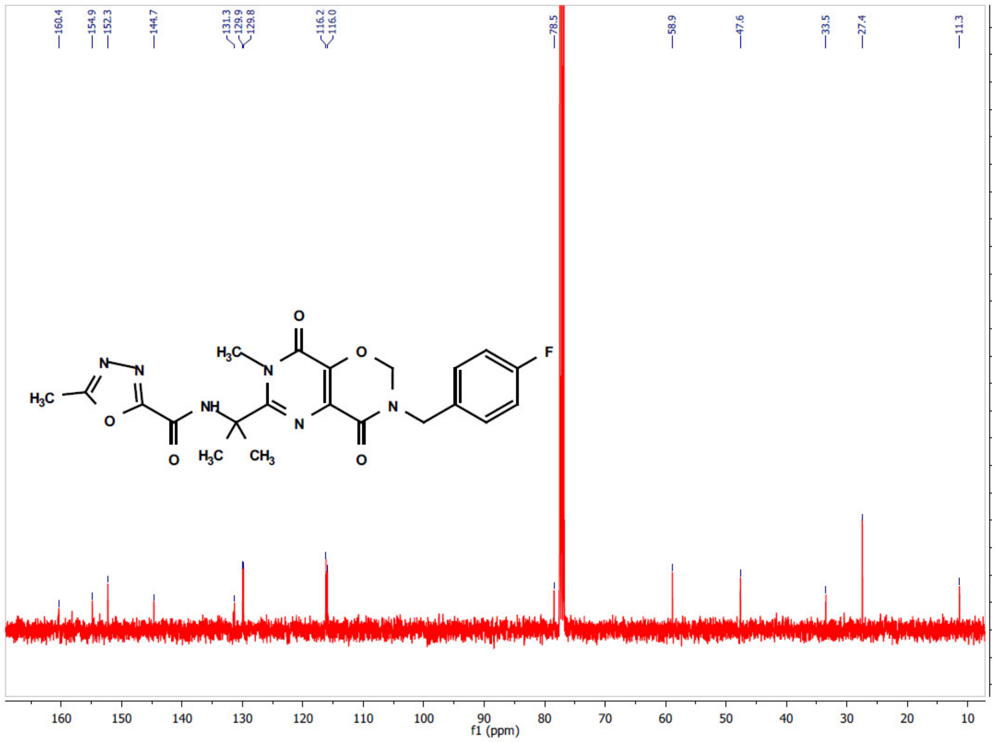

Figure S32. ^{13}C NMR of **22** in $CDCl_3$.

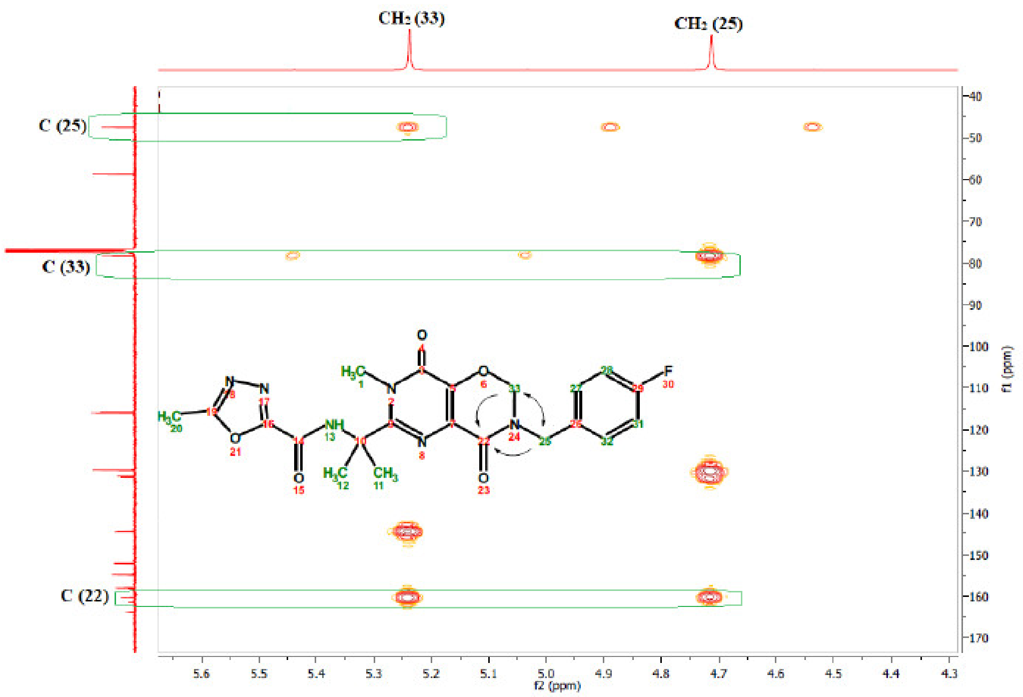

Figure S33. 2D NMR (HMBC) spectrum of **22** in $CDCl_3$.

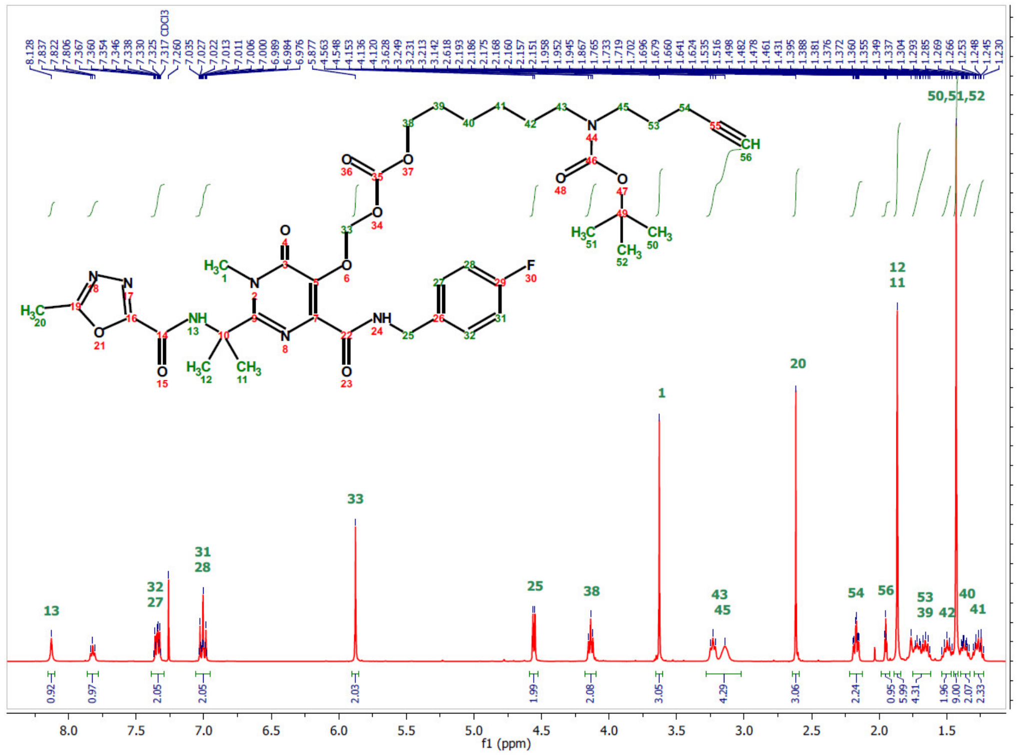

Figure S34. ^{1}H NMR of **23** in $CDCl_3$.

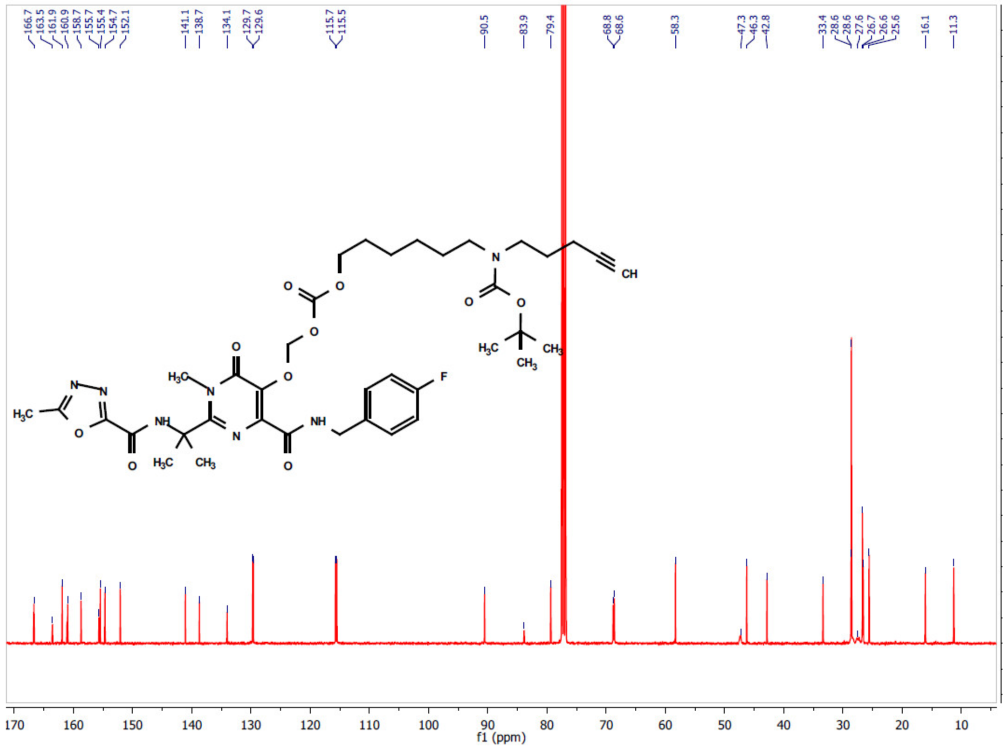

Figure S35. ^{13}C NMR of **23** in $CDCl_3$.

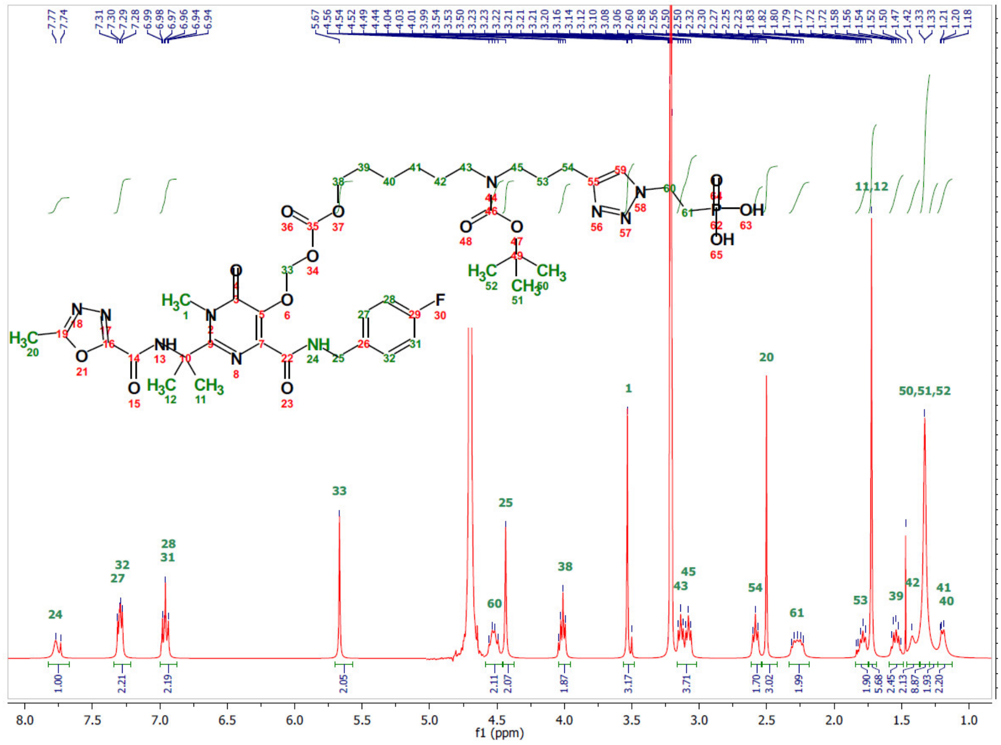

Figure S36. ^{1}H NMR of **24** in MeOD.

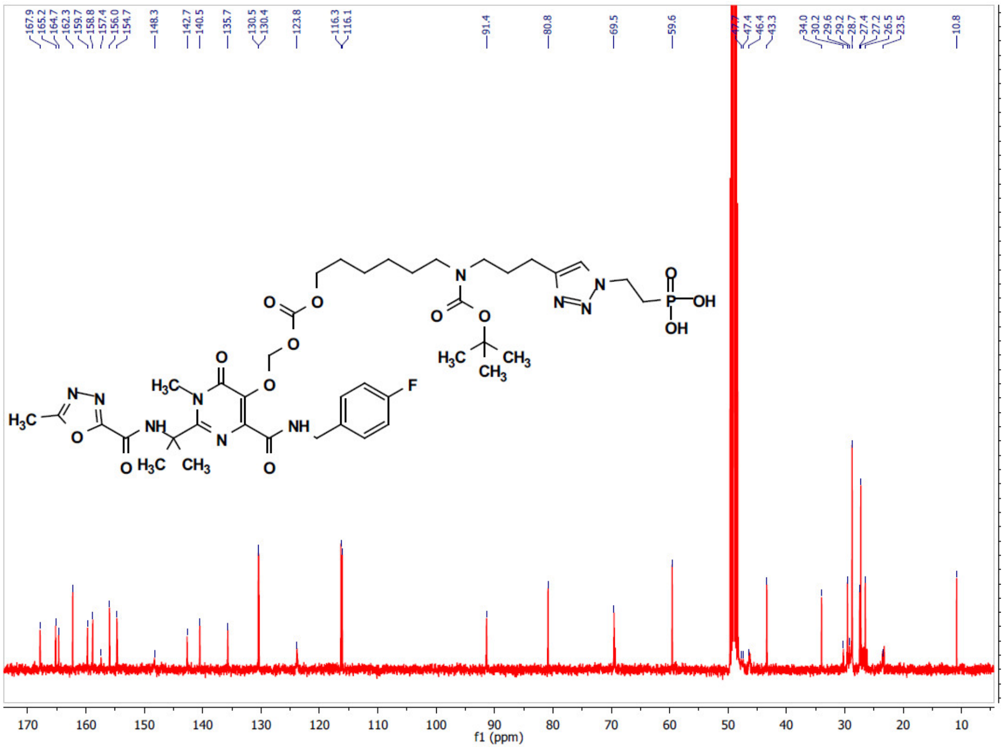

Figure S37. ^{13}C NMR of **24** in MeOD.

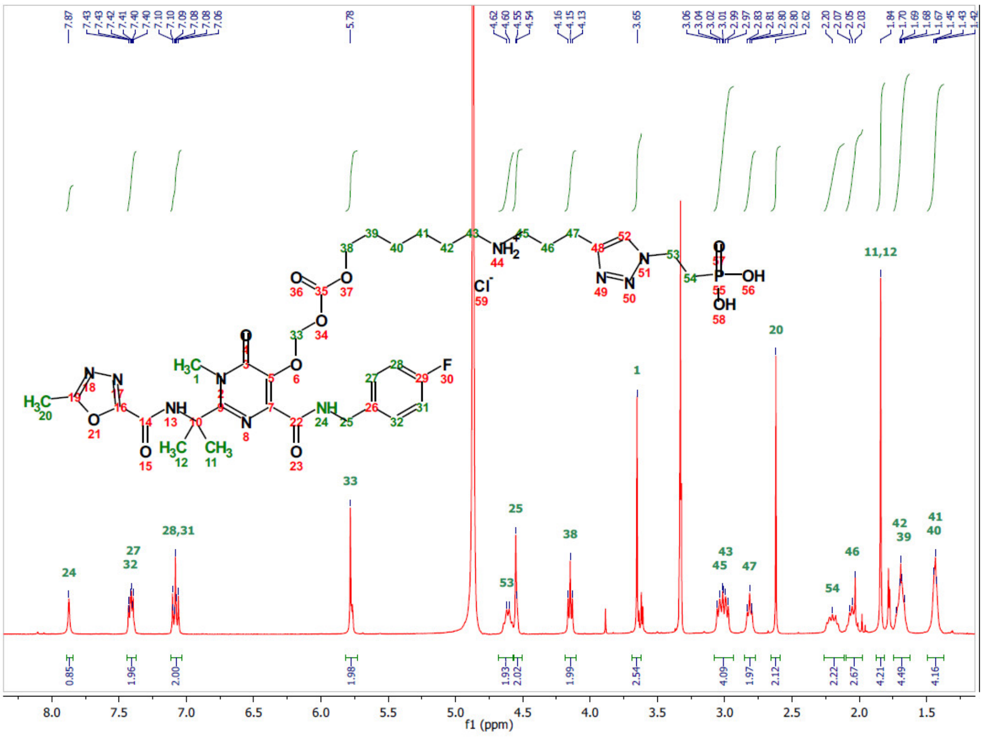

Figure S38. ^{1}H NMR of prodrug **25** in MeOD.

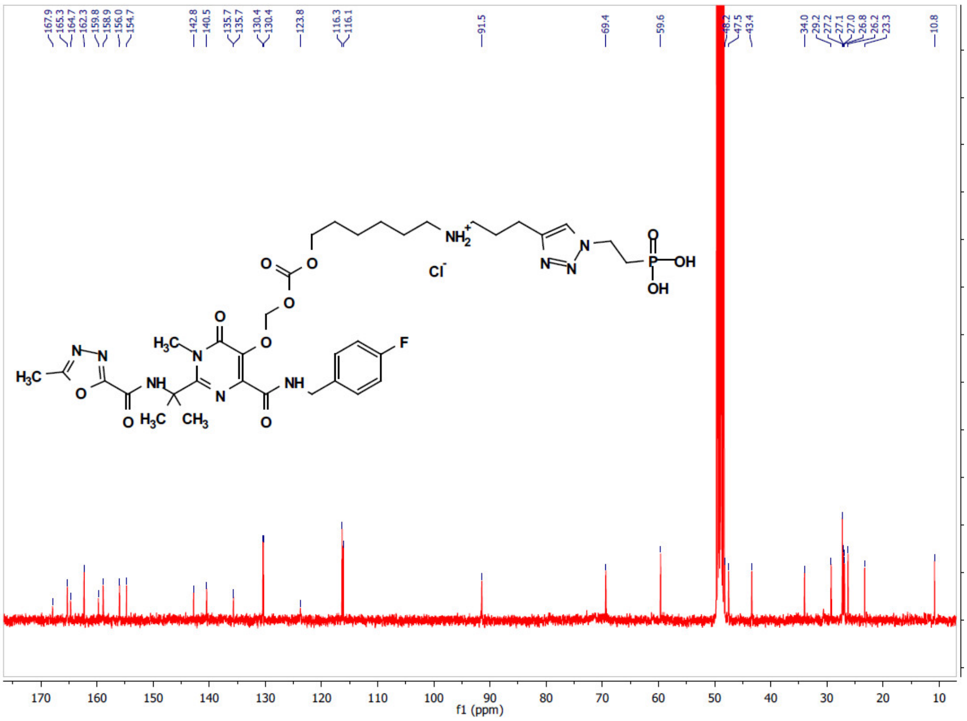

Figure S39. ^{13}C NMR of prodrug **25** in MeOD.

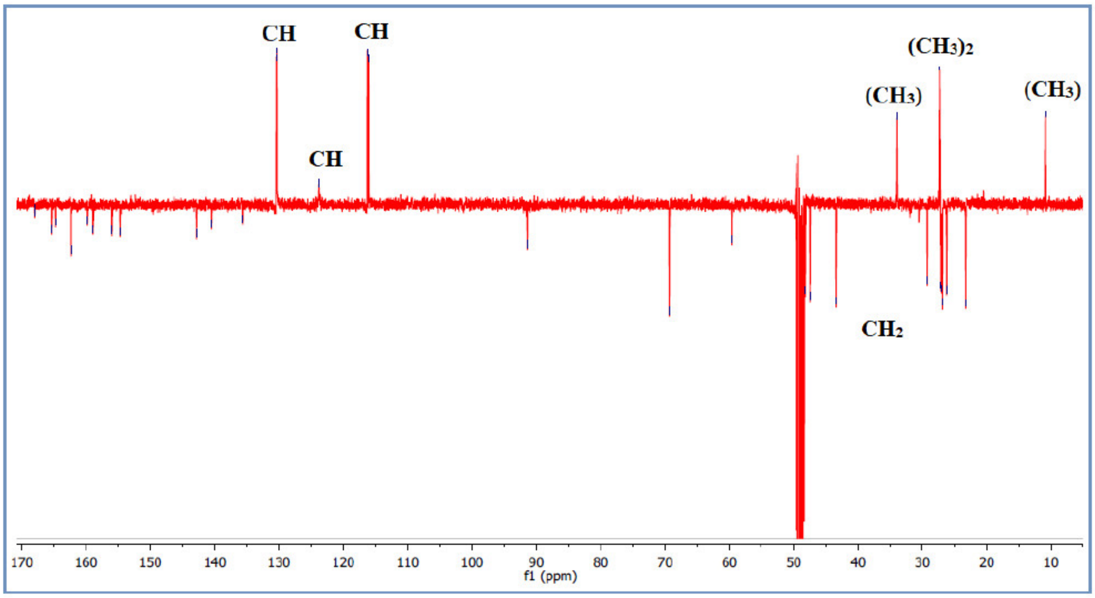

Figure S40. DEPT 135 ^{13}C NMR of prodrug **25** in MeOD.

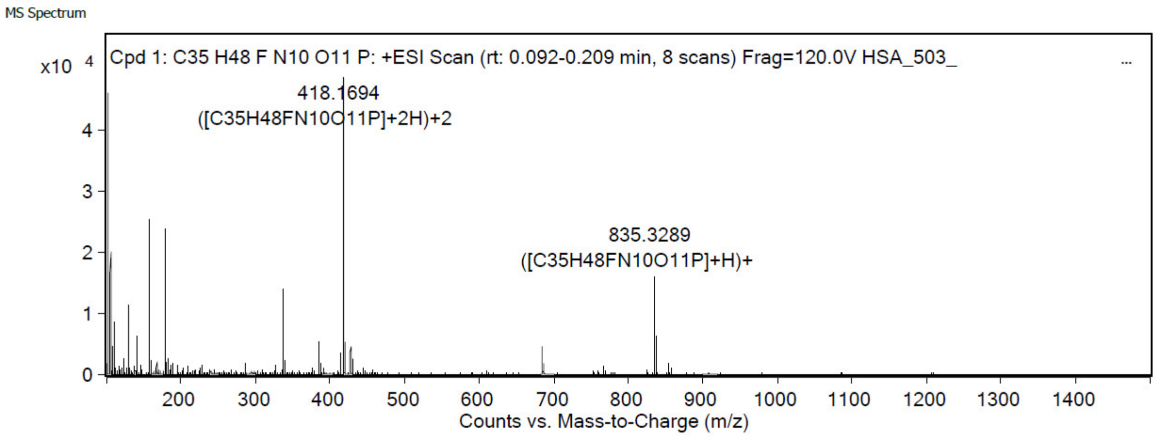

Figure S41. HRMS of prodrug **25**.

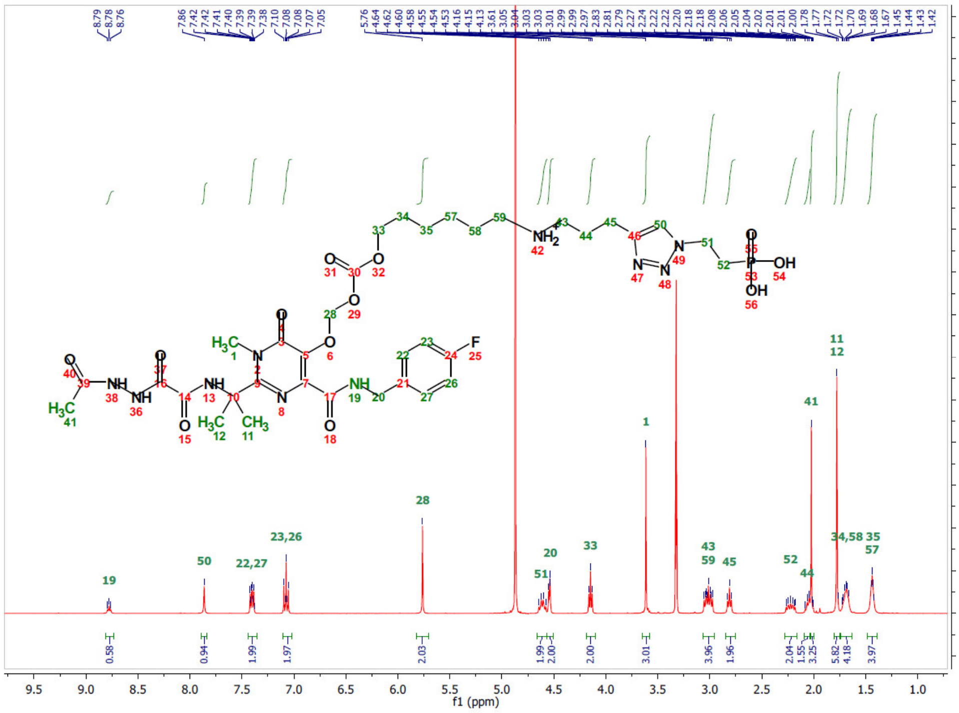

Figure S 42. ^{1}H NMR of **26** in MeOD.

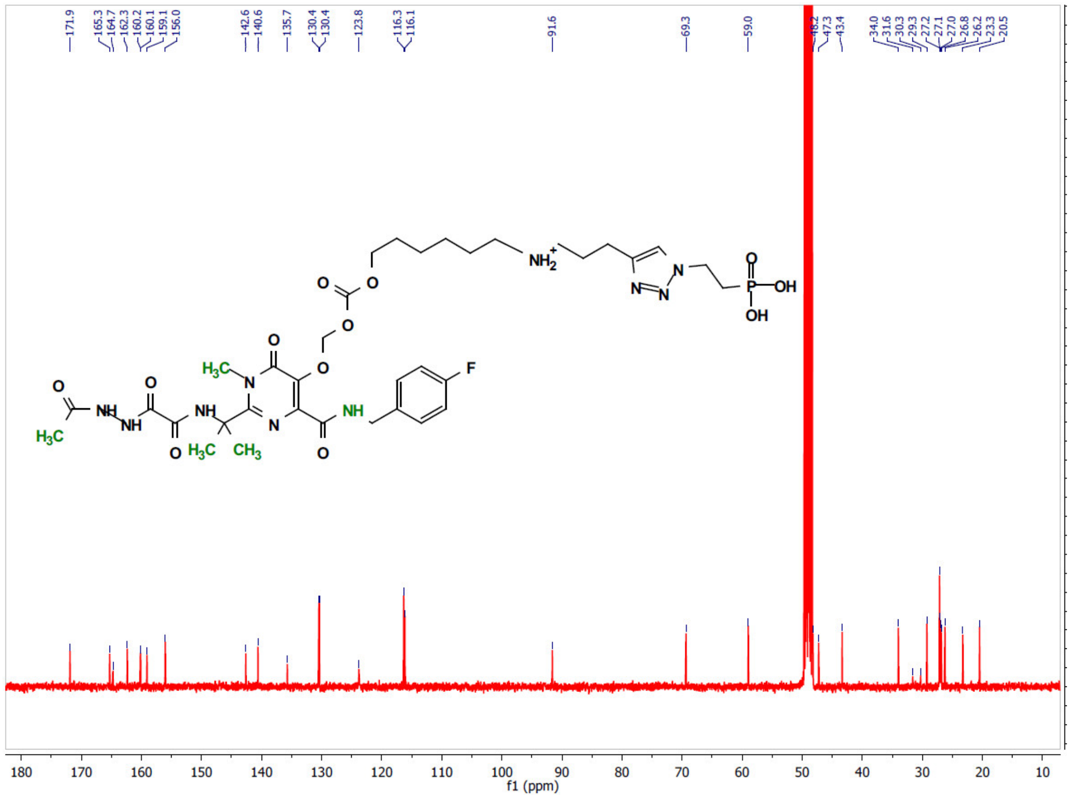

Figure S43. ^{13}C NMR of **26** in MeOD.

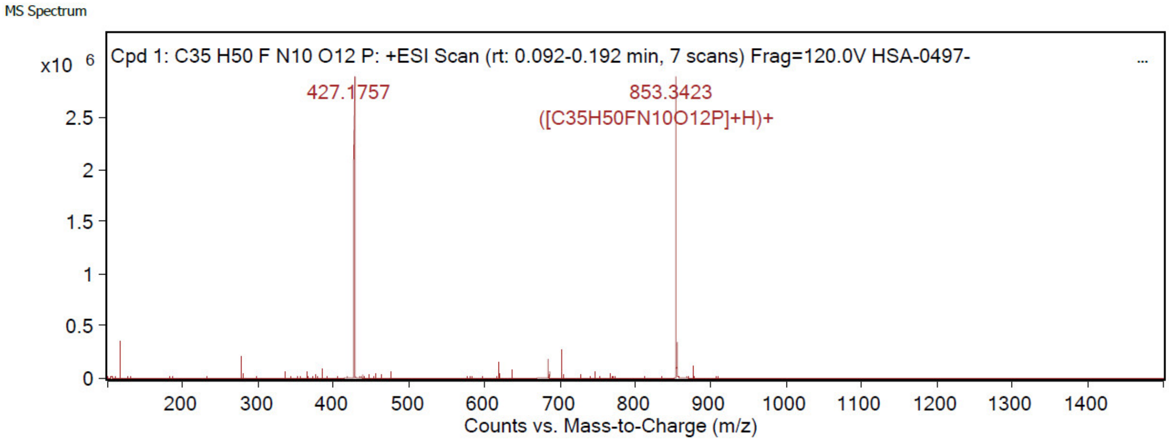

Figure S44. HRMS of **26**.
